# Supplementary material for: Amyloid-polysaccharide interfacial coacervates as therapeutic materials
Source: Nat Commun. 2023 Apr 3;14:1848. doi: 10.1038/s41467-023-37629-z (PMC10070338; doi:10.1038/s41467-023-37629-z)
Supplement: Supplementary file 1 — Supplementary Information [file 41467_2023_37629_MOESM1_ESM.pdf]

Supplementary Information

Amyloid-polysaccharide interfacial coacervates as therapeutic materials

Mohammad Peydayesh[1], Sabrina Kistler[2], Jiangtao Zhou[1], Viviane Lutz-Bueno[1,3], Francesca Damiani Victorelli[1], Andréia Bagliotti Meneguin[4], Larissa Spósito[4,5], Tais Maria Bauab[5], Marlus Chorilli[4] and Raffaele Mezzenga[1,2*]

[1] ETH Zurich, Department of Health Sciences and Technology, 8092 Zurich, Switzerland

[2] ETH Zurich, Department of Materials, 8093 Zurich, Switzerland

[3] Paul Scherrer Institute PSI, 5232 Villigen, Switzerland

[4] Department of Drugs and Medicines, School of Pharmaceutical Sciences, São Paulo State University, 14800-903, Araraquara, Sao Paulo, Brazil

[5] Department of Biological Sciences, School of Pharmaceutical Sciences, São Paulo State University, 14800-903, Araraquara, Sao Paulo, Brazil

E-mail: raffaele.mezzenga@hest.ethz.ch

Supplementary Tab. 1. WAXS results.

**Name**	p_1 **(Å^{-1})**	d_1 **(Å)**	p_2 **(Å^{-1})**	d_2 **(Å)**
AF	0.62	10.11	1.41	4.45
HA	0.74	8.53	1.42	4.43
AF + HA	0.65	9.71	1.44	4.37

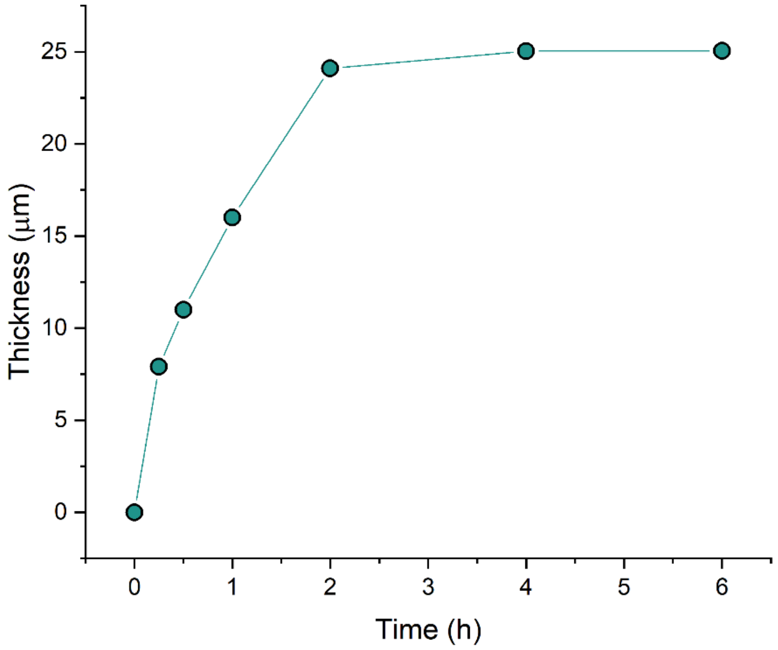

Supplementary Fig. 1. Effect of coacervation time on film thickness.

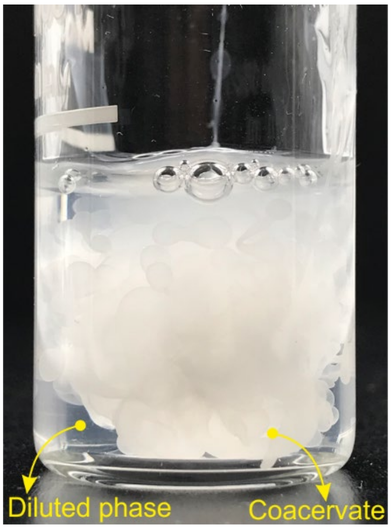

Supplementary Fig. 2. Bulk coacervation of AF (2 wt.%) and HA (1 wt.%) at pH 3. The two resulting phases after coacervation, i.e., coacervate and diluted phases, are visible.

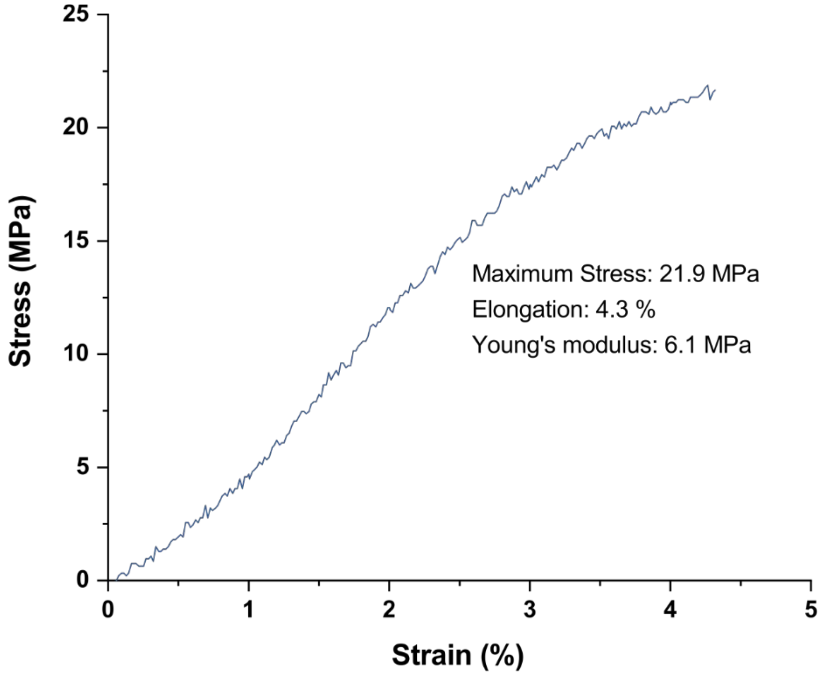

Supplementary Fig. 3. Mechanical properties of AF-HA films.

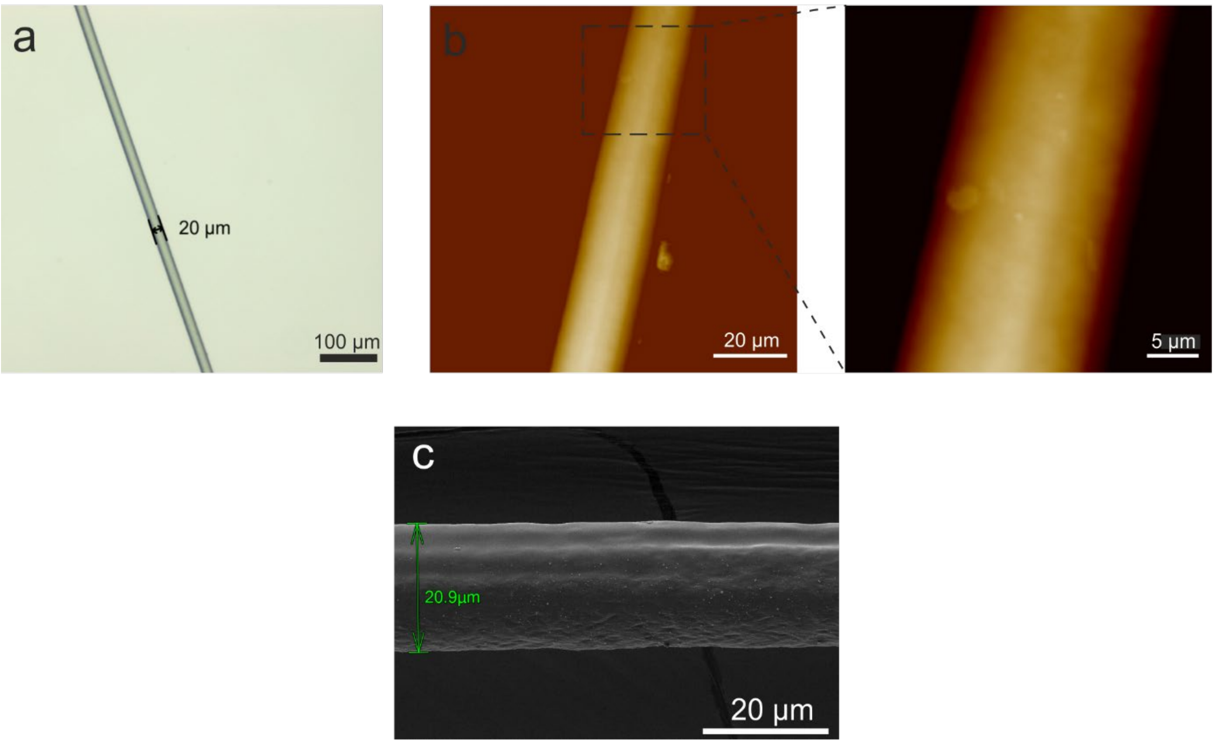

Supplementary Fig. 4. AF-HA fibers characterization by **a)** optical microscopy, **b)** AFM and **c)** SEM. Different microscopy experiments were repeated at least three times and representative images were shown.

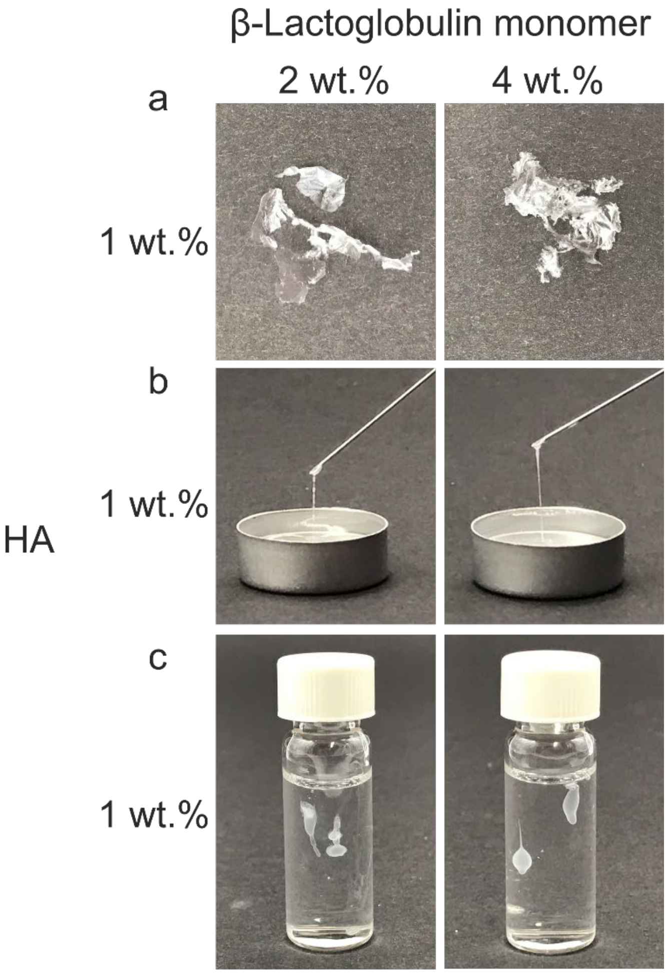

Supplementary Fig. 5. Photographs of a) films, b) fibers and c) capsules formed by coacervation of β-lactoglobulin monomer and HA at optimum pH of 3.

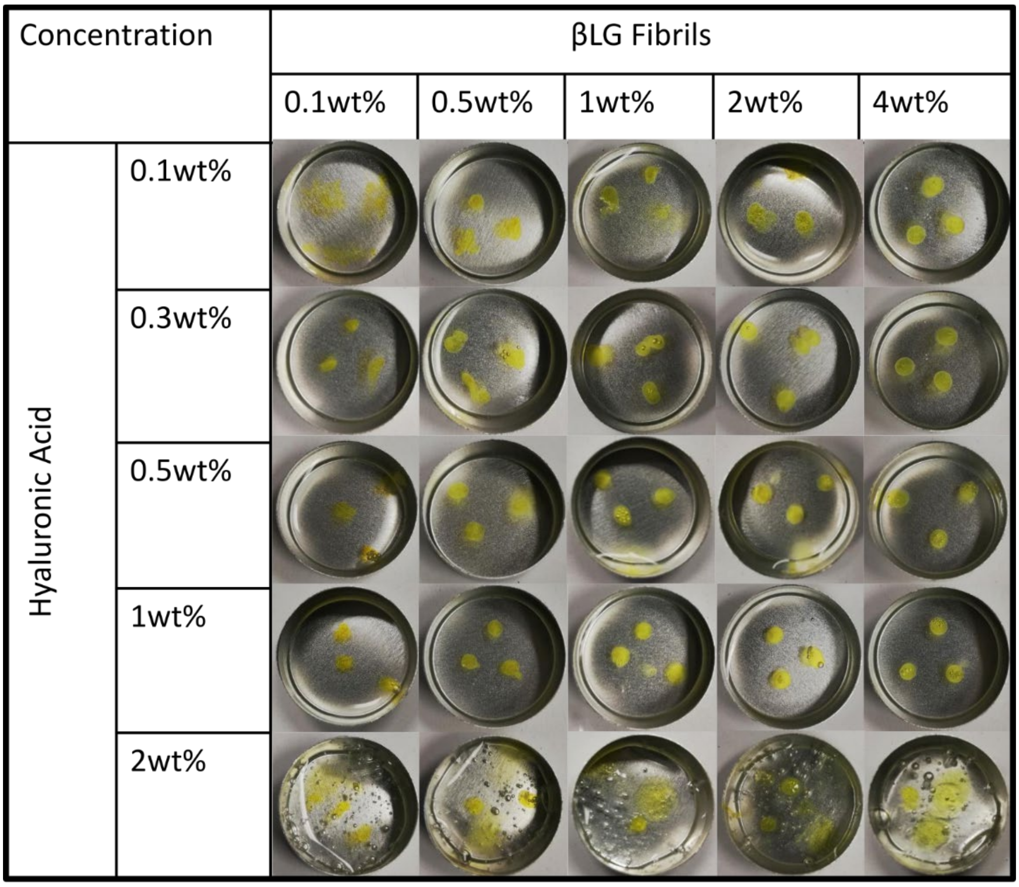

Supplementary Fig. 6. Images of AF-HA capsules in different concentrations (AF as the inner phase and HA as the outer phase). For better visualization, the inner phase was stained with curcumin.

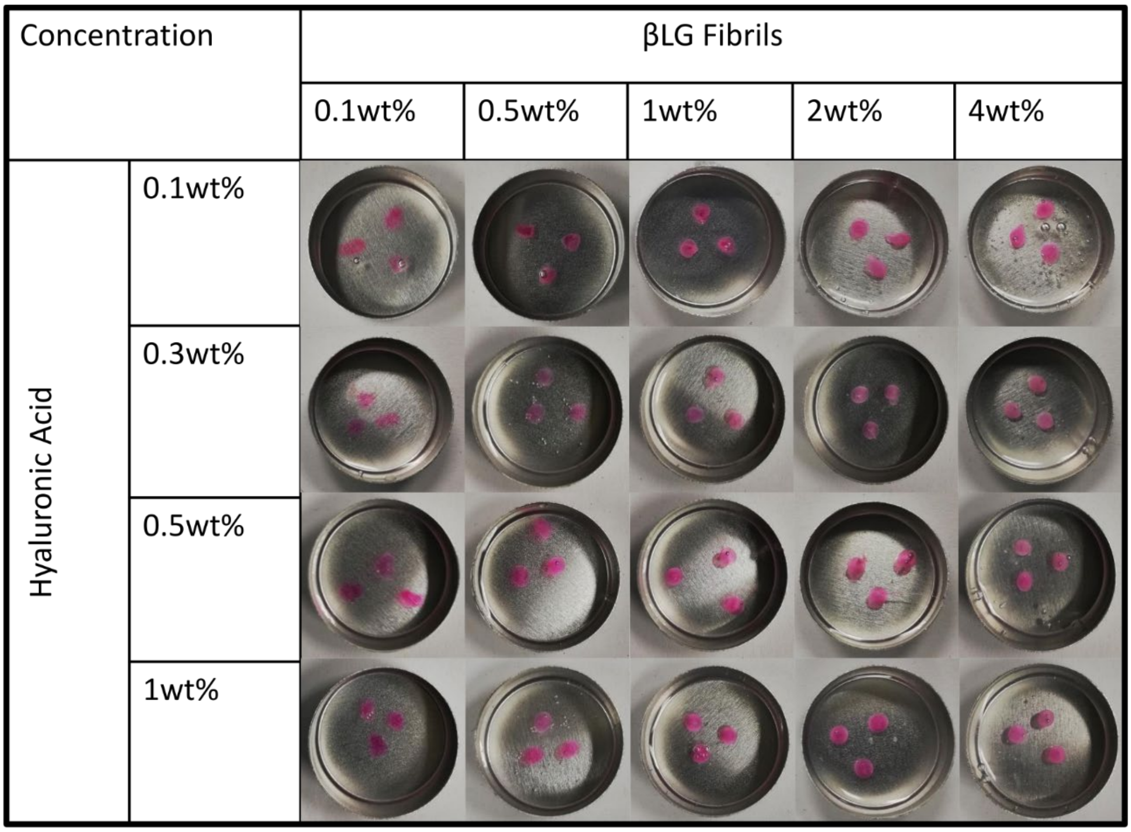

Supplementary Fig. 7. Images of HA-AF capsules in different concentrations (HA as the inner phase and AF as the outer phase). For better visualization, the inner phase was stained with acid fuchsin.

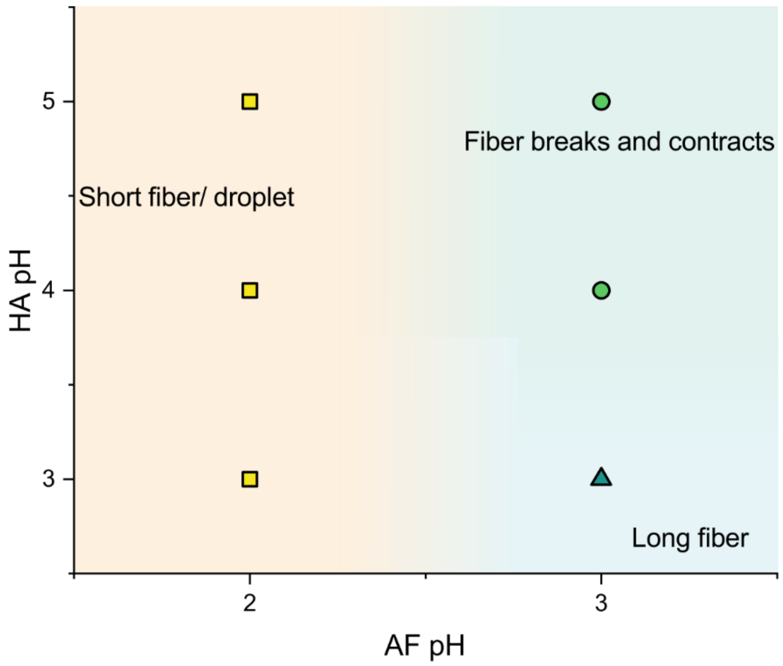

Supplementary Fig. 8. Concentration phase diagram for producing fibers (AF as the inner phase and HA as the outer phase).

pH		βLG Fibrils	
		2	3
Hyaluronic Acid	3	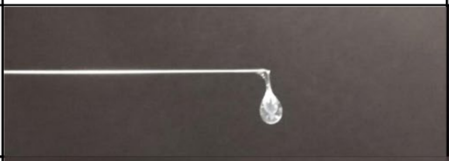	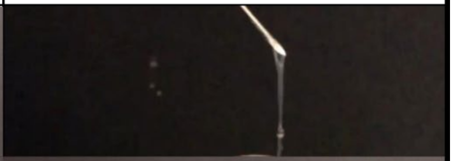
	4	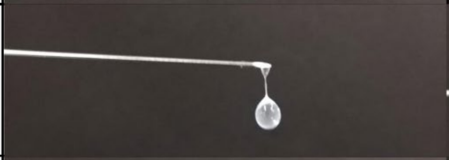	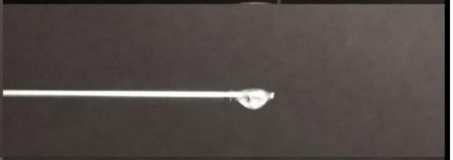
	5	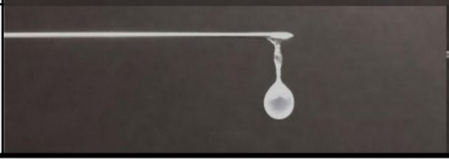	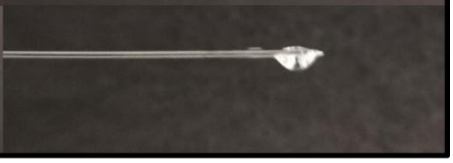

Supplementary Fig. 9. Images of AF-HA fibers in different concentrations (AF as the inner phase and HA as the outer phase).

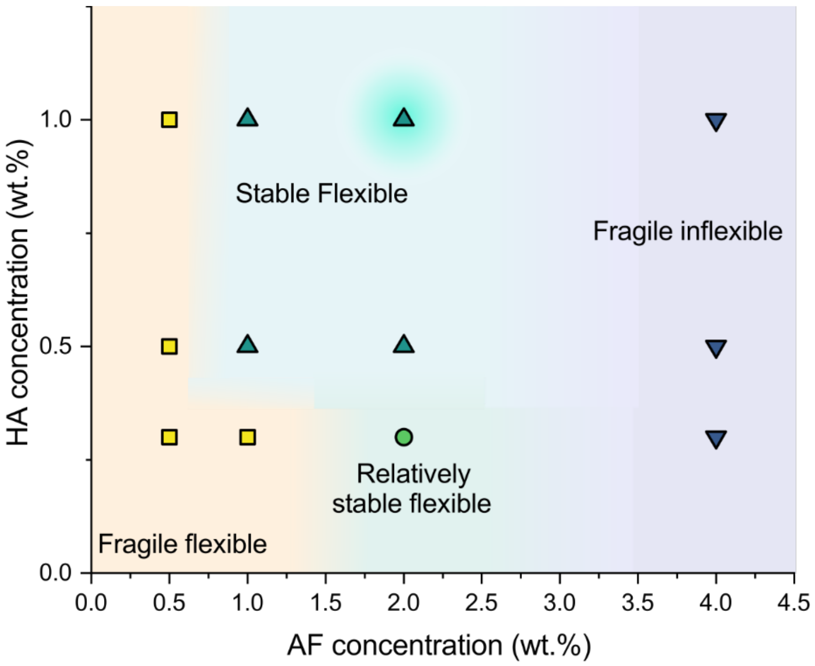

Supplementary Fig. 10. Concentration phase diagram for producing films (AF as the top phase and HA as the bottom phase).

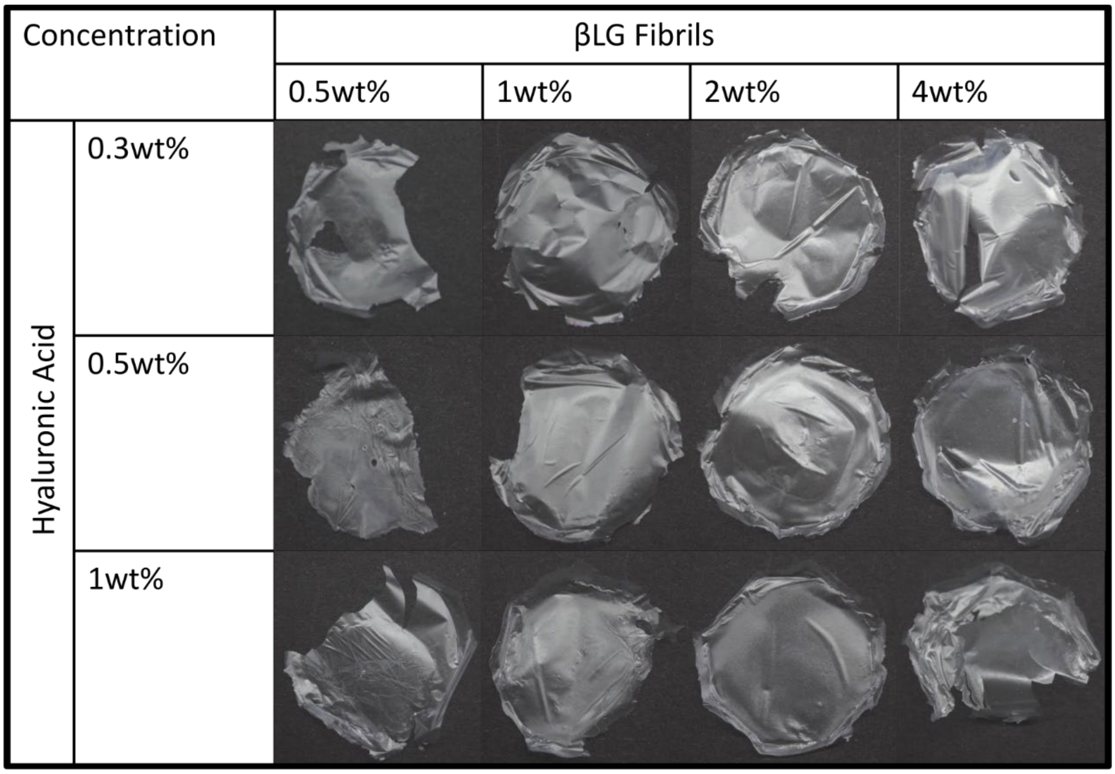

Supplementary Fig. 11. Images of AF-HA films in different concentrations (AF as the top phase and HA as the bottom phase).

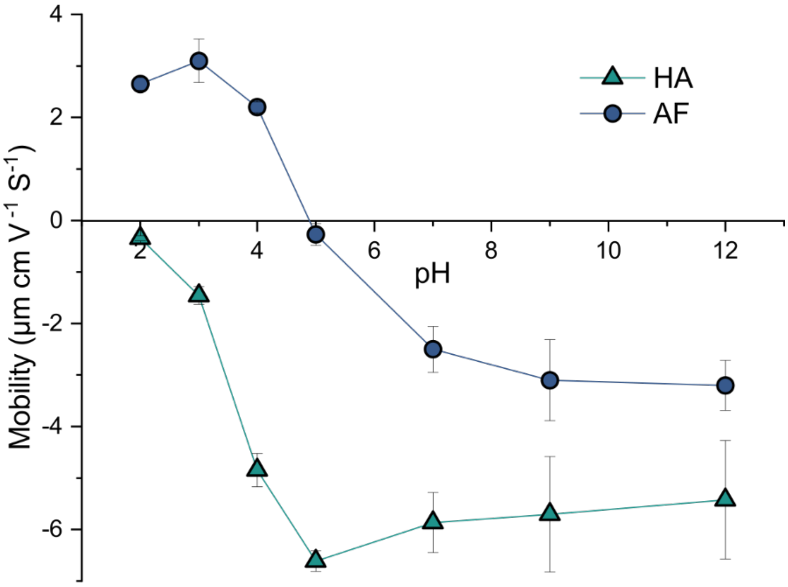

Supplementary Fig. 12. Electrophoretic mobility of AF and HA. Mean values ± SD (Number of replicates, n = 3).

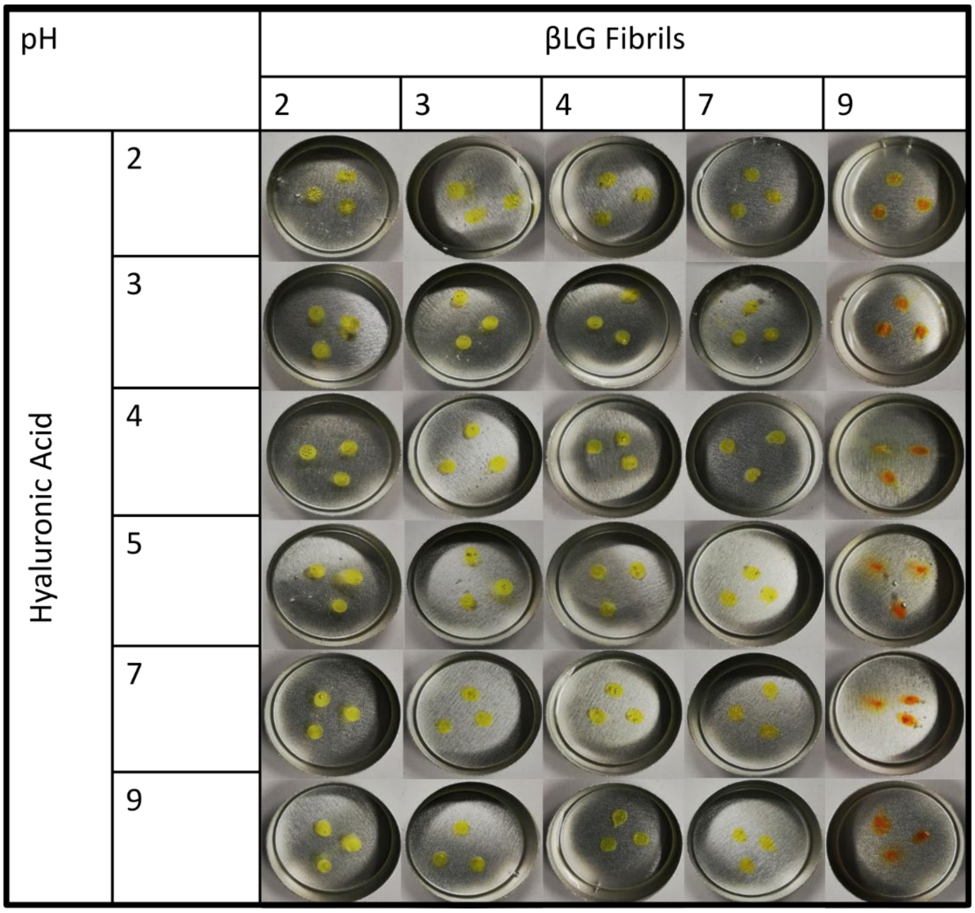

Supplementary Fig. 13. Images of AF-HA capsules in different pHs (AF as the inner phase and HA as the outer phase). For better visualization, the inner phase was stained with curcumin.

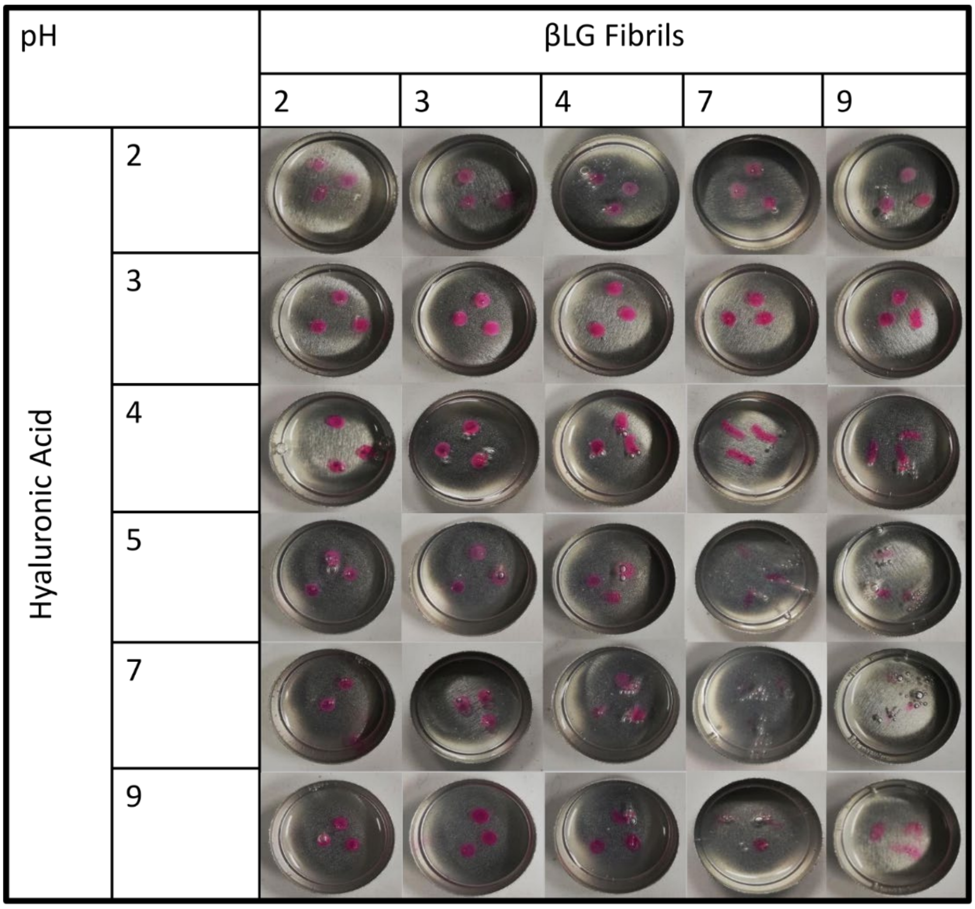

Supplementary Fig. 14. Images of HA-AF capsules in different pHs (HA as the inner phase and AF as the outer phase). For better visualization, the inner phase was stained with acid fuchsin.

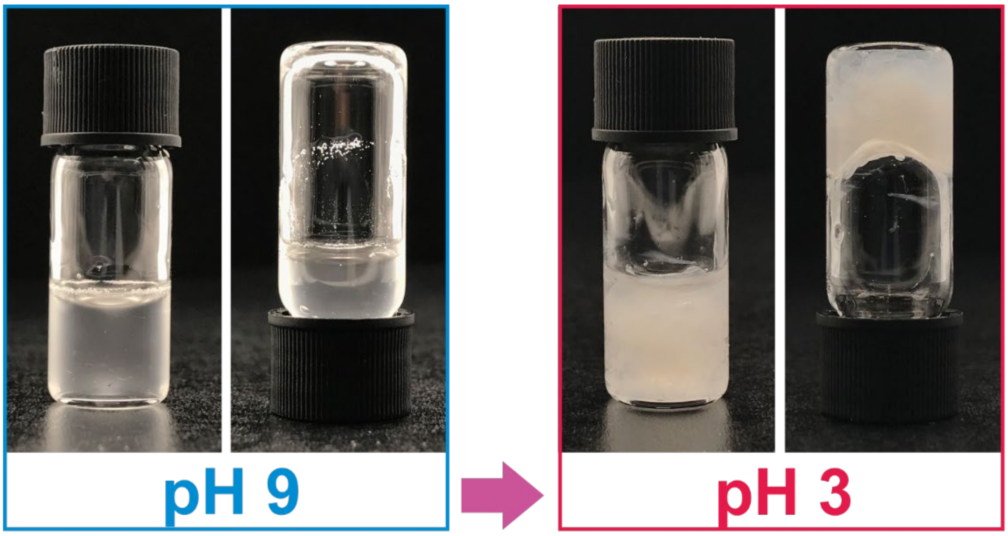

Supplementary Fig. 15. The role of electrostatic interaction in bulk coacervation of AF (2 wt.%) and HA (1 wt.%).

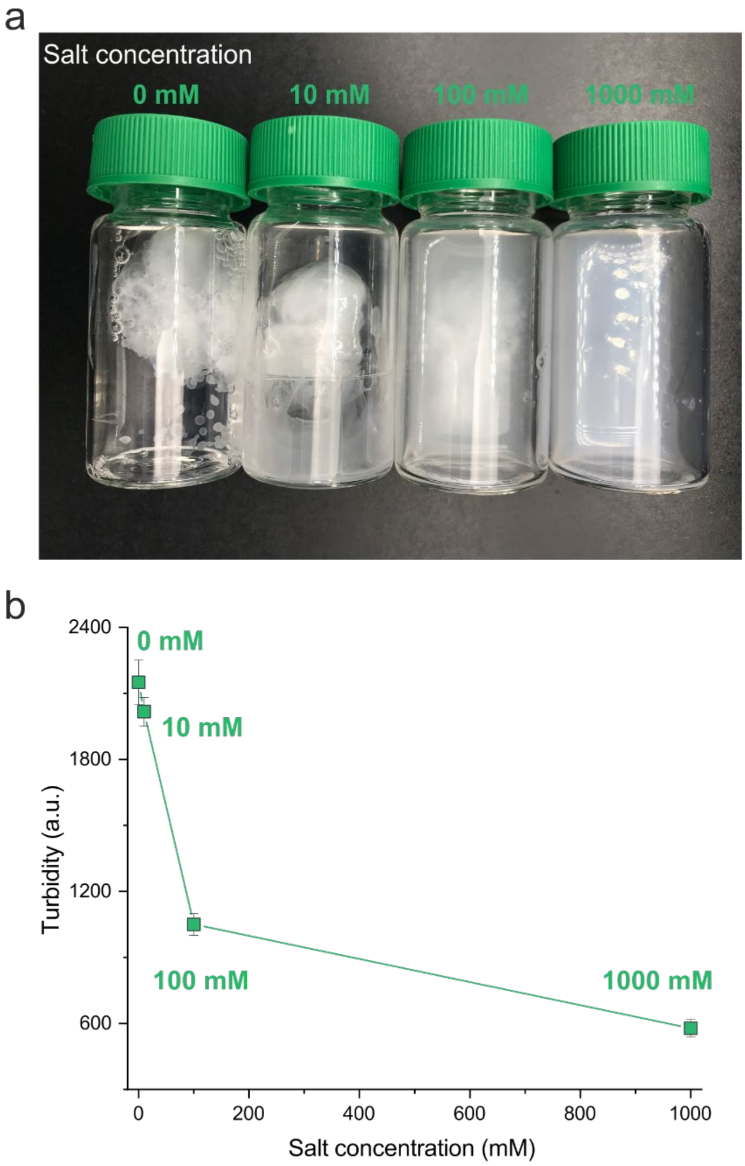

Supplementary Fig. 16. The ionic strength effect on coacervation of AF (2 wt.%) and HA (1 wt.%) at pH 3. a) The visual appearance of coacervation samples in different salt concentrations. b) Turbidity of samples as a function of salt concentration. Mean values ± SD (Number of replicates, n = 3).

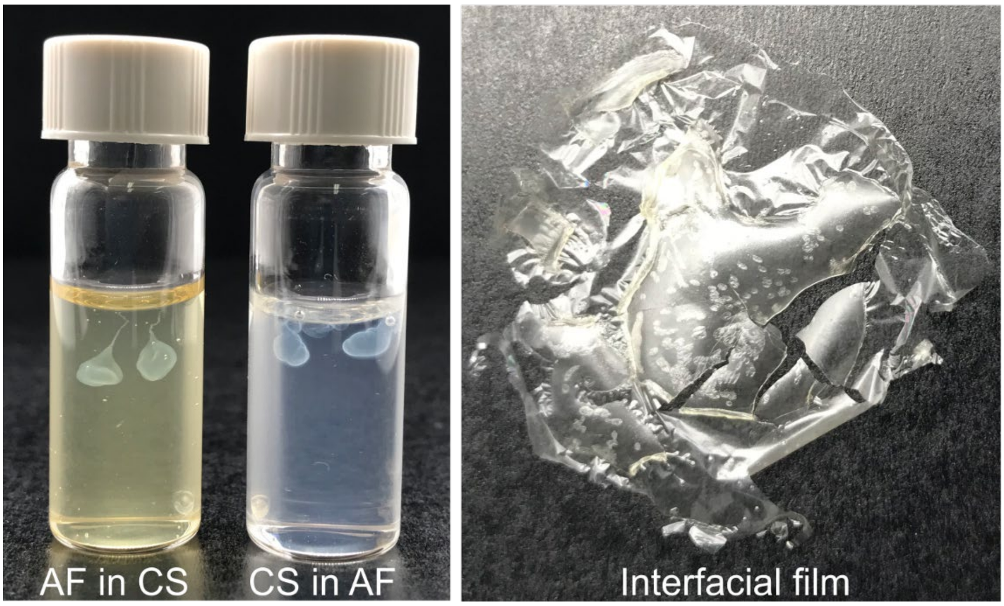

Supplementary Fig. 17. Coacervation of anionic AF (2 wt.%) and cationic CS (2 wt.%) at pH 8.

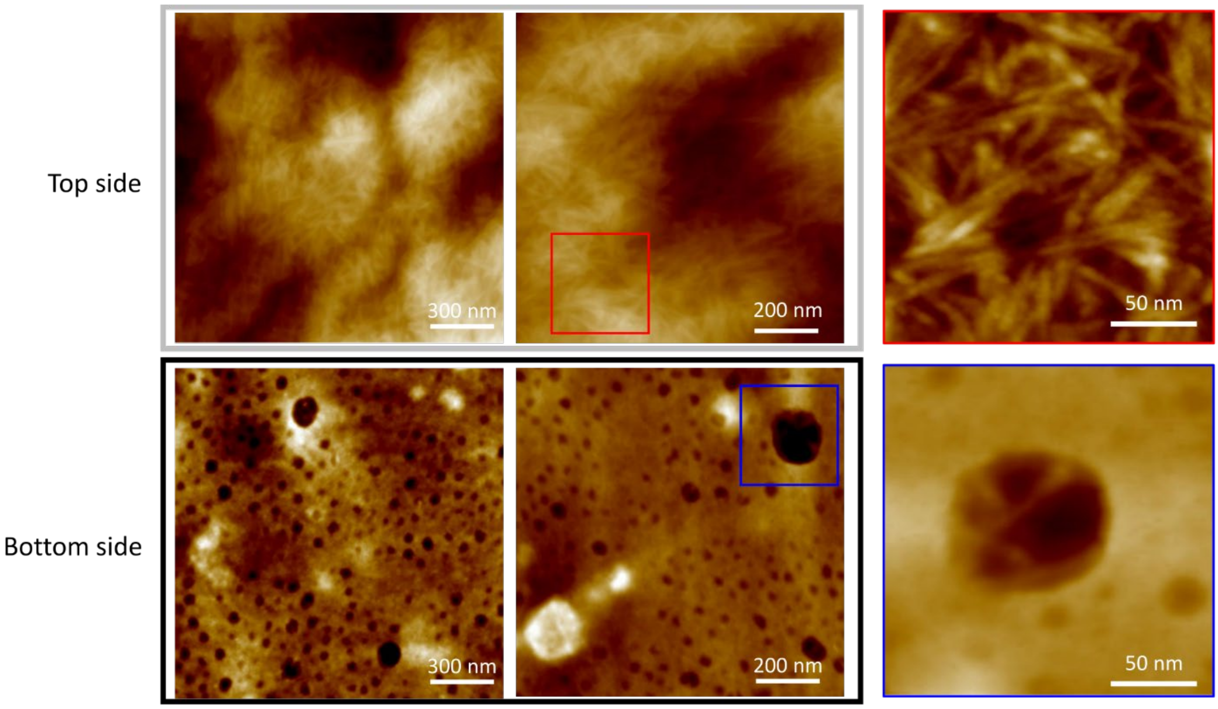

Supplementary Fig. 18. AFM Images of top and bottom surface of HA-AF film. The AFM experiment was repeated at least three times and representative images were shown.

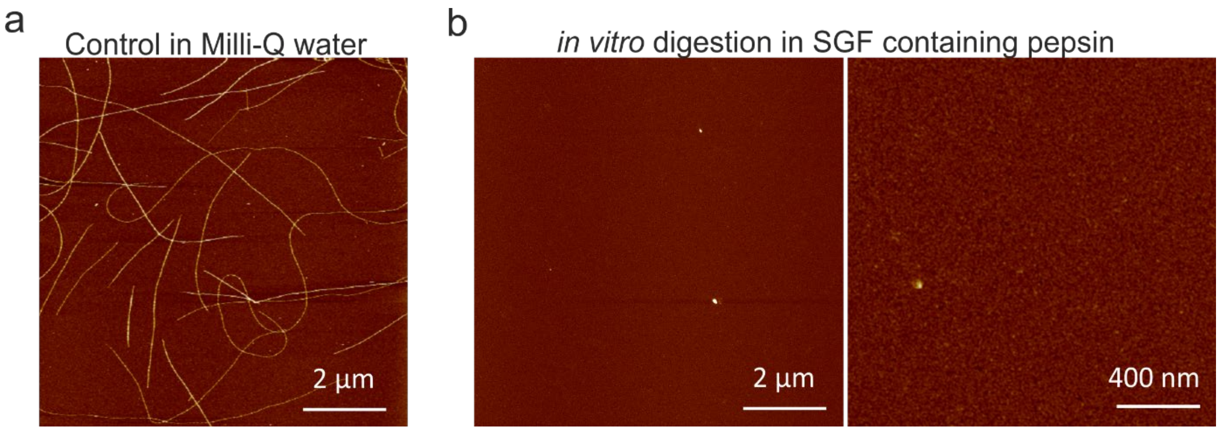

Supplementary Fig. 19. *in vitro* digestion of AF released from AF-HA capsules. a) Control in Milli-Q water. b) *in vitro* digestion in SGF containing pepsin. The AFM experiment was repeated at least three times and representative images were shown.

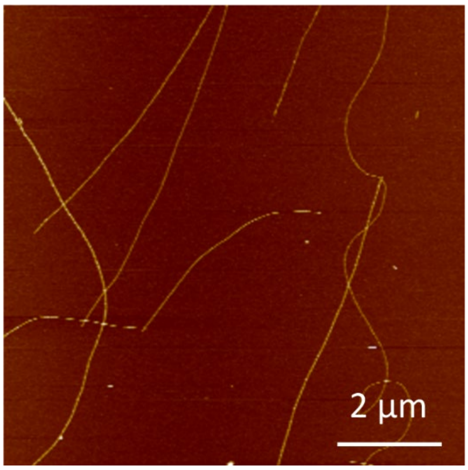

Supplementary Fig. 20. AFM image of β-lactoglobulin AF. The AFM experiment was repeated at least three times and representative images were shown.

Supplementary results on water purification application of prepared coacervates

Supplementary Fig. 21a and b demonstrate the water purification capability of AF-filled capsules and coacervate film to remove gold from the water. After treatment with capsules and film, the initial gold concentration of 234 ppm dropped to 37 and 5.7 ppm, indicating removal efficiencies of 84.2 and 97.6%, respectively. It is well known that protein nanofibrils are excellent adsorbents for heavy metals, thanks to their high surface-to-volume aspects, facilitating the metal-ligand chelation[1]. Additionally, as presented in Supplementary Fig. 21b, while the capsules entrapped the gold ions inside them in the AF phase, the gold ions were reduced to the elemental gold on the surface of the coacervate film, highlighting the possibility of one-step water purification and precious metal recovery.

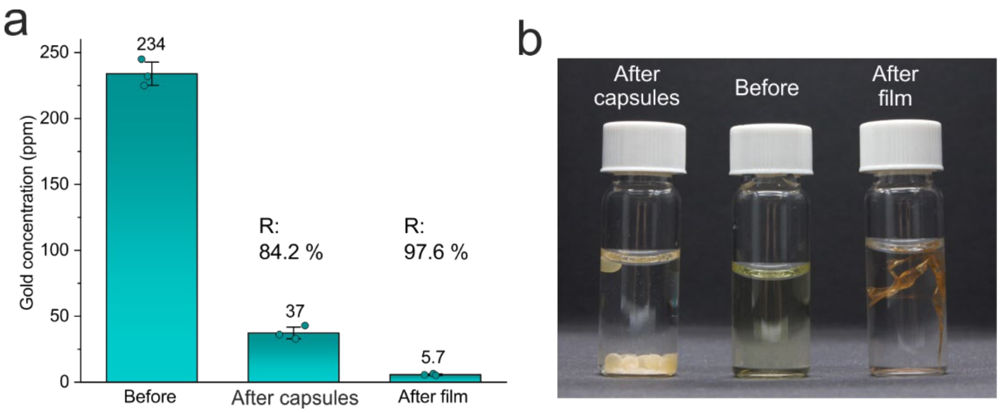

Supplementary Fig. 21. AF-HA coacervate water purification application. **a)** Water purification performance of AF-HA films and capsules for gold removal. Mean values ± SD (Number of replicates, n = 3). **b)** Images of gold removal from water with AF-HA films and capsules.

Supplementary methods for water purification experiments

The coacervate capsules and films were prepared by using AF (2 wt.%) and (HA 1 wt.%) at pH 3. Then they were placed into 2 mL of around 250 ppm gold solutions. After 24 h, the concentration of gold before and after treatment was measured with flame atomic absorption spectrophotometry (AAS) (Varian, Agilent Technologies). Eventually, the removal efficiency

was calculated as $R\%=\frac{C_i-C_e}{C_i}\times 100$, where C_i and C_e (ppm) are the initial and equilibrium gold concentrations.

Supplementary References

1 Peydayesh, M. & Mezzenga, R. Protein nanofibrils for next generation sustainable water purification. *Nature Communications* **12**, 3248, doi:10.1038/s41467-021-23388-2 (2021).
