# Supplementary material for: Amyloid-polysaccharide interfacial coacervates as therapeutic materials
Source: Nat Commun. 2023 Apr 3;14:1848. doi: 10.1038/s41467-023-37629-z (PMC10070338; doi:10.1038/s41467-023-37629-z)
Supplement: Supplementary file 3 — Additional Supplementary Files [file 41467_2023_37629_MOESM3_ESM.pdf]

Description of Additional Supplementary Files

File Name: Supplementary Movie 1
Description: AF-HA coacervation to produce uniform fibers.
