# Supplementary material for: Amyloid-polysaccharide interfacial coacervates as therapeutic materials
Source: Nat Commun. 2023 Apr 3;14:1848. doi: 10.1038/s41467-023-37629-z (PMC10070338; doi:10.1038/s41467-023-37629-z)
Supplement: Supplementary file 5 — Reporting Summary [file 41467_2023_37629_MOESM5_ESM.pdf]

nature portfolio

Corresponding author(s): Raffaele Mezzenga

Last updated by author(s): 14.02.2023

Reporting Summary

Nature Portfolio wishes to improve the reproducibility of the work that we publish. This form provides structure for consistency and transparency in reporting. For further information on Nature Portfolio policies, see our Editorial Policies and the Editorial Policy Checklist.

Statistics

For all statistical analyses, confirm that the following items are present in the figure legend, table legend, main text, or Methods section.

n/a	Confirmed	
☐	☒	The exact sample size (*n*) for each experimental group/condition, given as a discrete number and unit of measurement
☐	☒	A statement on whether measurements were taken from distinct samples or whether the same sample was measured repeatedly
☐	☒	The statistical test(s) used AND whether they are one- or two-sided<br>*Only common tests should be described solely by name; describe more complex techniques in the Methods section.*
☒	☐	A description of all covariates tested
☒	☐	A description of any assumptions or corrections, such as tests of normality and adjustment for multiple comparisons
☐	☒	A full description of the statistical parameters including central tendency (e.g. means) or other basic estimates (e.g. regression coefficient) AND variation (e.g. standard deviation) or associated estimates of uncertainty (e.g. confidence intervals)
☐	☒	For null hypothesis testing, the test statistic (e.g. *F*, *t*, *r*) with confidence intervals, effect sizes, degrees of freedom and *P* value noted<br>*Give P values as exact values whenever suitable.*
☒	☐	For Bayesian analysis, information on the choice of priors and Markov chain Monte Carlo settings
☒	☐	For hierarchical and complex designs, identification of the appropriate level for tests and full reporting of outcomes
☒	☐	Estimates of effect sizes (e.g. Cohen's *d*, Pearson's *r*), indicating how they were calculated

Our web collection on statistics for biologists contains articles on many of the points above.

Software and code

Policy information about availability of computer code

Data collection	No Software was used.
Data analysis	ImageJ®software was used to analyze the lesions.

For manuscripts utilizing custom algorithms or software that are central to the research but not yet described in published literature, software must be made available to editors and reviewers. We strongly encourage code deposition in a community repository (e.g. GitHub). See the Nature Portfolio guidelines for submitting code & software for further information.

Data

Policy information about availability of data

All manuscripts must include a data availability statement. This statement should provide the following information, where applicable:

- Accession codes, unique identifiers, or web links for publicly available datasets
- A description of any restrictions on data availability
- For clinical datasets or third party data, please ensure that the statement adheres to our policy

The authors declare that all data supporting the findings of this study are available within the paper and its Supplementary Information files or available from the corresponding author upon request.

Human research participants

Policy information about studies involving human research participants and Sex and Gender in Research.

Reporting on sex and gender	N/A
Population characteristics	N/A
Recruitment	N/A
Ethics oversight	N/A

Note that full information on the approval of the study protocol must also be provided in the manuscript.

Field-specific reporting

Please select the one below that is the best fit for your research. If you are not sure, read the appropriate sections before making your selection.

☒ Life sciences ☐ Behavioural & social sciences ☐ Ecological, evolutionary & environmental sciences

For a reference copy of the document with all sections, see nature.com/documents/nr-reporting-summary-flat.pdf

Life sciences study design

All studies must disclose on these points even when the disclosure is negative.

Sample size

32 rats
The sample size is based on the review paper "Questões em bioestatística: o tamanho da amostra" (MOURÃO JÚNIOR, 2009) and on previous experiments carried out by our research group. Since the percentage of resilient/susceptible animals varies between the different shipments from the bioterium, the number of animals used in the study will be based on previous laboratory work that indicates an approximate percentage of 45% of resilient/susceptible animals. The sample number will be n =8 per group, with 4 groups, totaling 32 animals, based on the available literature, about experiments involving similar tests (SPÓSITO et al., 2019; AMARAL et. al. 2017).

AMARAL, Thiago Yamamoto et al. Antimicrobial and anti-inflammatory activities of Apis mellifera honey on the Helicobacter pylori infection of Wistar rats gastric mucosa. Food Science and Technology, v. 37, p. 34-41, 2017.
MOURÃO JÚNIOR, C. A. Questões em bioestatística: o tamanho da amostra. Revista Interdisciplinar de Estudos Experimentais, v. 1, n.1, p. 26-28, 2009.
SPÓSITO, Larissa et al. In vitro and in vivo anti-Helicobacter pylori activity of Casearia sylvestris leaf derivatives. Journal of ethnopharmacology, v. 233, p. 1-12, 2019.

Data exclusions	No data exclusion
Replication	8 animals per group. All attempts at replication were successful.
Randomization	The rats were randomly assigned to 4 groups: Positive control (PC), omeprazole (OME) 40 mg/Kg, AF-HA coacervate microparticles (MP) 33.3 mg/kg, and negative control (NC).
Blinding	The investigators were blinded to group allocation during the data collection/or analysis of the stomach lesions

Reporting for specific materials, systems and methods

We require information from authors about some types of materials, experimental systems and methods used in many studies. Here, indicate whether each material, system or method listed is relevant to your study. If you are not sure if a list item applies to your research, read the appropriate section before selecting a response.

Materials & experimental systems

n/a	Involved in the study
☒	☐ Antibodies
☒	☐ Eukaryotic cell lines
☒	☐ Palaeontology and archaeology
☐	☒ Animals and other organisms
☒	☐ Clinical data
☒	☐ Dual use research of concern

Methods

n/a	Involved in the study
☒	☐ ChIP-seq
☒	☐ Flow cytometry
☒	☐ MRI-based neuroimaging

Animals and other research organisms

Policy information about studies involving animals; ARRIVE guidelines recommended for reporting animal research, and Sex and Gender in Research

Laboratory animals	We used 32 heterogeneous male rats, Rattus norvegicus (Wistar), at the age of 7 weeks.
Wild animals	No wild animals were used in the study.
Reporting on sex	Male<br>Although the development of ulcers is not sex-dependent,the incidence of this disease is higher among male individuals (Kavitt et al., 2019; Xie et al., 2022; Ren et al., 2022). Hence we carried out the experiments only with male rats. Despite this, we believe that it is important to study ulcer in female rats in future studies as well.<br><br>Kavitt, R. T., et al. Diagnosis and Treatment of Peptic Ulcer Disease. The American Jornal of Medicine, 132, p. 447-456, 2019.<br>Xie, X., et al. The global, regional and national burden of peptic ulcer disease from 1990 to 2019: a population-based study. BMC Gastroenterology, 22, 58, 2022.<br>Ren, J., et al. The global burden of peptic ulcer disease in 204 countries and territories from 1990 to 2019: a systematic analysis for the Global Burden of Disease Study 2019. International Journal of Epidemiology, p. 1666-1676, 2022.
Field-collected samples	No field collected samples were used in the study.
Ethics oversight	All the protocols were approved by the ethics committee of the School of Pharmaceutical Sciences - Araraquara - SP - Brazil for animal care and use (CEUA/FCFAr: 13/2021) and were conducted according to the principles of the National Council for Animal Experiments Control (CONCEA), based on the National Institutes of Health (NIH) Guidelines for the Care and Use of Laboratory Animals

Note that full information on the approval of the study protocol must also be provided in the manuscript.
